# Validation of the estimated glomerular filtration rate equation for Japanese children younger than 2 years

**DOI:** 10.1007/s10157-021-02165-x

**Published:** 2022-01-01

**Authors:** Yoshimitsu Gotoh, Osamu Uemura, Naoya Fujita, Yuko Hamasaki, Masataka Honda, Kenji Ishikura, On behalf of the Pediatric CKD Study Group in Japan in conjunction with the Committee of Measures for Pediatric CKD of the Japanese Society of Pediatric Nephrology, Yoshimitsu Gotoh, Osamu Uemura, Naoya Fujita, Yuko Hamasaki, Masataka Honda, Kenji Ishikura

**Affiliations:** 1 Department of Pediatric Nephrology, Japanese Red Cross Aichi Medical Center Nagoya Daini Hospital, 2-9 Myoken-cho Showa-ku, Nagoya-shi, Aichi 466-8650 Japan; 2 Department of Pediatrics, Ichinomiya Medical Treatment and Habilitation Center, 1679-2 Tomida-nagaresuji, Ichinomiya-shi, Aichi 494-0018 Japan; 3 Department of Pediatric Nephrology, Aichi Children’s Health and Medical Center, 7-426 Morioka-cho, Obu-shi, Aichi 474-8710 Japan; 4 Department of Nephrology, Toho University Faculty of Medicine, 6-11-1 Oomori Nishi Ota-ku, Tokyo, 143-8541 Japan; 5 Department of Nephrology, Tokyo Metropolitan Children’s Medical Center, 2-8-29 Musasidai, Futyu-shi, Tokyo 183-8561 Japan; 6 Department of Pediatrics, Kitasato University Hospital, 1-15-1 Kitasato Minami-ku, Sagamihara-shi, Kanagawa 252-0375 Japan

**Keywords:** Validation, Estimated glomerular filtration rate, Children under 2 years of age, Japanese

## Abstract

**Background:**

We have developed a simple and easy method of estimating the glomerular filtration rate (eGFR) of serum creatinine in Japanese children (eGFR_Uemura_). The eGFR equation is for children aged 2–18 years. Therefore Uemura et al. developed an equation for children younger than 2 years (eGFR_under 2_). The aim of the present study was to validate this new equation.

**Methods:**

We collected the data of 13 patients from previous studies and compared the results of eGFR_under 2_, eGFR_Uemura_, and updated eGFR developed by Schwartz (eGFR_Schwartz_) with measured GFR using mean error (ME), root mean square error (RMSE), *P*_30_ and Bland–Altman analysis.

**Results:**

The ME of eGFR_under 2_, eGFR_Uemura_ and eGFR_Schwartz_ were 2.3 ± 15.9, 7.7 ± 14.5, and 16.0 ± 18.2 ml/min/1.73m^2^, respectively. The RMSEs were 15.5, 15.9, and 49.6, respectively. The *P*_30_ values were 76.9%, 76.9%, and 53.8%, respectively. The graph of Bland–Altman bias analysis showed fan-shape. The eGFR_under 2_ equation was the most accurate in the three equations.

**Conclusion:**

The eGFR_under 2_ equation was useful for Japanese children younger than 2 years.

## Introduction

The gold standard for evaluation of renal function is inulin clearance (Cin). However, the procedure of Cin is complicated and difficult, especially in younger children and/or patients with bladder dysfunction. Therefore, various simple and easy methods to determine the estimated GFR (eGFR) have been developed. Recently, the most known equation for eGFR used in children is the updated Schwartz (eGFR_Schwartz_) equation [1]. However, Uemura et al. reported that the eGFR_Schwartz_ equation is not applicable for Japanese children [2]. Therefore, an original equation for eGFR (eGFR_Uemura_) was developed using serum creatinine (Cr) in Japanese children [3], and its accuracy was validated [4]. However, the equation was for children aged 2–18 years. Therefore, an equation for children younger than 2 years was then developed by multiplying eGFR_Uemura_ by a coefficient (0.107 × ln(age[month]) + 0.656) [5]. In this study, we aimed to validate eGFR_under 2_ equation.

## Material and methods

### Study population

We extracted the data of patients under 2 years of age from three studies. The first was 7 of 174 patients’ data when the eGFR_Uemura_ equation was created [3]. The second was 8 of 140 patients’ data when the accuracy of the eGFR_Uemura_ equation was validated [4]. The third was 1 of 59 patients’ data validated the safety of Inulide**®** for Japanese children (in press) (Table 1). All data were collected from pediatric patients with chronic kidney disease (CKD) in clinical need of Cin. Finally, we used data from 13 patients after excluding patients (Table1).

**Table 1:** Characteristics of the 13 patients included in this study

Characteristics	Median (IQR)	*n*
Age (months)	17.0 (10.0–20.5)	
Gender		
Male		11
Female		2
CKD stage		
Stage 1		2
Stage 2		4
Stage 3		6
Stage 4		1
Renal abnormality		
Congenital anomalies of the kidney and urinary tract		6
Solitary kidney		3
Reflux nephropathy		2
Hydronephrosis		1
Small kidney		1

*CKD* chronic kidney disease, *IQR* interquartile range

The measured GFR (mGFR) for each patient was obtained using Cin. The procedure is described in a previous report [6]. Cin values were measured in the same way in three studies.

The calculate method of eGFR_under 2_ is as follows:

1. The reference serum Cr level (ref Cr) is shown by the following two equations of body length (*x*):
  males: ref Cr = − 1.259x^5^ + 7.815*x*^4^ – 18.57*x*^3^ + 21.39*x*^2^ – 11.71*x* + 2.628
  females: ref Cr = − 4.536*x*^5^ + 27.16*x*^4^ – 63.47*x*^3^ + 72.43*x*^2^ – 40.06*x* + 8.778
2. Provisional GFR = 110.2 × (ref Cr/ patient’s serum Cr) + 2.93
3. *R* = 0.107 × ln [age (months)] + 0.656
4. eGFR under 2 years of age (eGFR_under 2_) = *R* × provisional GFR

### Exclusion criteria and cases excluded

The exclusion criteria were as follows:

1. Primary diseases including severe obstructive uropathy, infection during treatment, inflammatory disease, dehydration, neuromuscular disease, severe cardiac, hepatic, or pancreatic disease, and/or endocrine disease, including thyroid impairment.
2. Cases in which the ratios of inulin excretion and intravenous inulin administration were < 0.5, or > 1.5, during the measurement of Cin. We determined the dose of intravenous inulin by assuming that the blood concentration was constant during testing. Ratios of inulin excretion and intravenous inulin that were not within 0.5 and 1.5 may have been due to failure to collect all urine.
3. Cases in which the measured GFR (mGFR) was > 150 ml/min/1.73m^2^; pediatric patients with CKD due to a hyperfiltration disease such as diabetic nephropathy are rare. We were only interested in cases in which GFR was < 120 ml/min/1.73m^2^. Therefore, we excluded cases in which mGFR was > 150 ml/min/1.73m^2^.

### Statistical analysis

To validate eGFR _under 2_ equation, we used four methods as follows:

1. Mean error (ME): to evaluate the mean difference between each value for eGFR and mGFR values
2. Root mean square error (RMSE): to evaluate the precision of eGFR and mGFR values.
3. *P*_30_ (the percentage of eGFR value within 30% of mGFR): to evaluate the accuracy of eGFR and mGFR values.
4. Bland–Altman analysis (difference versus average): to determine agreement with the estimation.

We also compared these statistical results of eGFR_under 2_ equations, with the results of eGFR_Uemura_, and eGFR_Schwartz_.

The formula for eGFR _Uemura_ equation is mentioned above in the calculation method of eGFR_under 2_ from (1) to (2). First, calculate the reference Cr level as described (1), and then the eGFR_Uemura_ from reference Cr and patient’s serum Cr using the method described in (2).

The updated Schwartz equation is as follows: eGFR (ml/min/1.73 m^2^) = 0.413 × body length (cm)/serum Cr value (mg/dL) by enzymatic Cr determination in children aged 1–16 years [1]. Therefore, in the second analysis, the data of three patients under 1 year of age were excluded. Finally, data from 10 patients were used for comparison between eGFR_under 2_ and eGFR_Schwartz_.

All analyses were performed using GraphPad Prism for Mac OS X (version 7.0).

## Results

### Characteristics of the study population (Table 1)

**Fig. 1:**
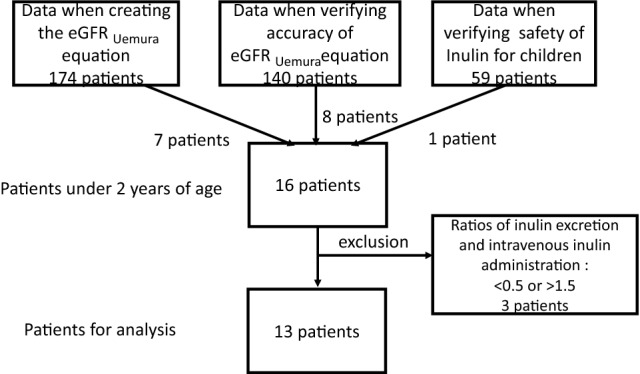
Flow chart of included data

Data of 6 patients were extracted from the three studies. Data of three patients were excluded because the ratios of urinary inulin excretion to intravenous inulin administration were < 0.5 or > 1.5. None of the patients were included in the 1 or 3 exclusion criteria. Finally, 13 patients’ data (2 female, median 17.0 months of age [interquartile range (IQR) 10.0–20.5 months, range 1–23 months]) were used for analysis (Fig. 1). The number of chronic kidney disease (CKD) stages 1, 2, 3, 4, and 5 were 2, 4, 6, 1, and 0, respectively. The number of renal abnormalities, congenital anomalies of the kidney and urinary tract (CAKUT), solitary kidney, reflux nephropathy, hydronephrosis, and small kidney were 6, 3, 2, 1, and 1, respectively.

### Result of statistical analysis

**Fig. 2:**
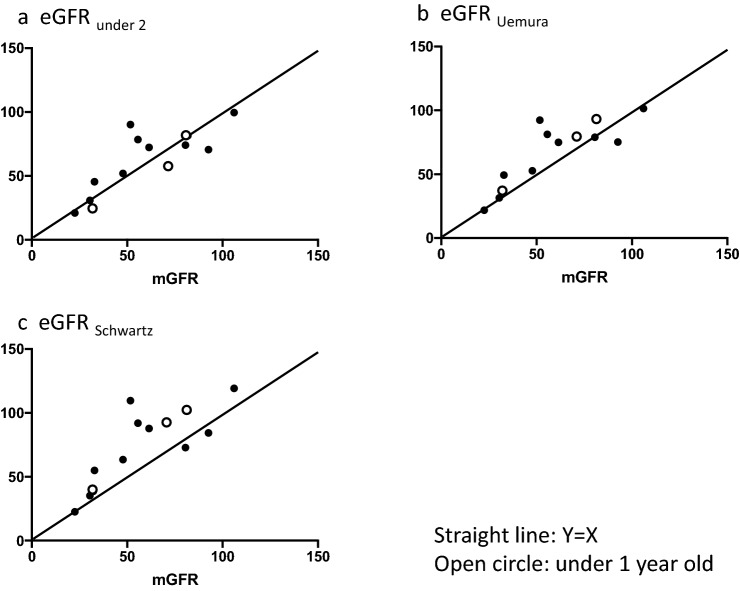
Scatter plot of mGFR versus each 3 eGFR equations. **a** eGFR_under 2_, **b** eGFR_Uemura_, **c** eGFR_Schwartz_. Straight line: *Y* = *X*, Open circle: data of children under 1-year-old

**Fig. 3:**
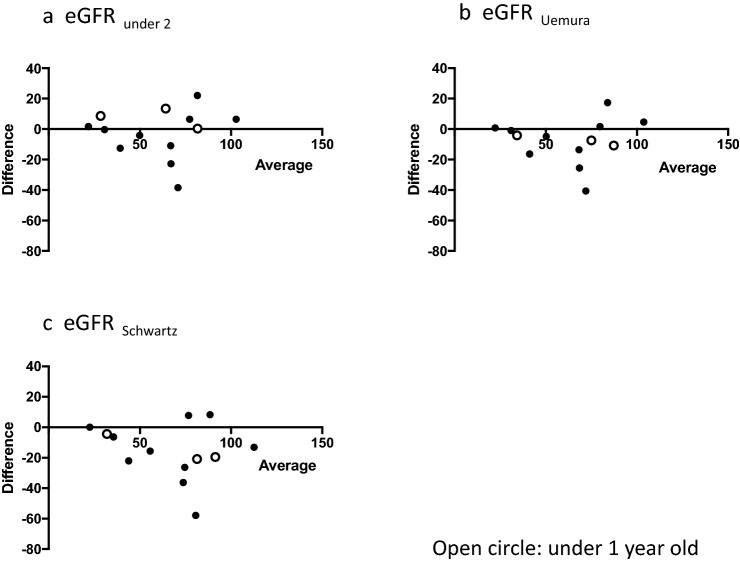
Bland–Altman plot. Difference versus average mGFR and each 3 eGFR equations. **a** eGFR_under 2_, **b** eGFR_Uemura_, **c** eGFR_Schwartz_. Open circle: data of children under 1 year old

Figure 2 shows a scatter plot of mGFR versus each of the three eGFR equations. The straight line shows the equivalent values of mGFR and eGFR. The open circles represent the patient data under 1 year of age. The scatter plots of eGFR_under 2_ and eGFR_Uemura_ seem to be similar. Figure 3 shows the Bland–Altman plot of the difference versus the average for both mGFR and each 3 eGFR equations.

Table 2a shows the numerical values for each examination in all 13 patients. The ME values for eGFR_under 2_, eGFR_Uemura_, and eGFR_Schwartz_ were 2.3 ± 15.9, 7.7 ± 14.5, and 16.0 ± 18.2 ml/min/1.73m^2^, respectively. The RMSE values were 15.5, 15.9, and 49.6 ml/min/1.73m^2^, respectively. The *P*_30_ values were 76.9%, 76.9%, and 53.8%, respectively. Table 2b shows the numerical values for the results of eGFR_under 2_ and eGFR_Schwartz_ in 10 patients aged between 1 and 2 years. The ME values for eGFR_under 2_, and eGFR_Schwartz_ were 5.3 ± 17.0 and 16.0 ± 20.7 ml/min/1.73m^2^, respectively. The RMSE values were 16.9 and 50.0 ml/min/1.73 m^2^, respectively. The *P*_30_ values were 70.0%, and 50.0%, respectively.

**Table 2:** Comparison of performance using three eGFR equations, eGFR_under 2_ equation, eGFR_Uemura_ equation and eGFR_Schwartz_

(a) Comparison of results with data from 13 patients			
	ME (bias) (ml/min/1.73m^2^) (95% CI)	RMSE (ml/min/1.73m^2^)	*P*_30_ (%)
eGFR_under2_	2.3 ± 15.9 (− 7.3 to 12.0)	15.5	76.9
eGFR_Uemura_	7.7 ± 14.5 (− 1.0 to 16.5)	15.9	76.9
eGFR_Schwartz_	16.0 ± 18.2 (5.0 to 27.0)	49.6	53.8

(b) Comparison of results of eGFR_under2_ and eGFR_Schwartz_ equation from 10 patients’ data between the ages of 1 and 2 years			
	ME (bias) (ml/min/1.73m^2^) (95% CI)	RMSE (ml/min/1.73m^2^)	*P*_30_ (%)
eGFR_under2_	5.3 ± 17.0 (− 6.9 to 17.4)	16.9	70.0
eGFR_Schwartz_	16.0 ± 20.7 (1.2 to 30.8)	50.0	50.0

*eGFR* estimated glomerular filtration rate, *ME* mean error, *P*_30_ the percentage of eGFR value within 30% of measured GFR, *RMSE* root mean square error

Figure 3 shows a scatter plot of the Bland–Altman analysis. In all three equations, the spread of the graph of the Bland–Altman analysis was fan-like shaped, with systematic error.

In the three equations, the values using eGFR_under 2_ equation showed the lowest value for ME and Bland–Altman analysis, and showed the larger values for P_30_ than that for eGFR_Schwartz_.

## Discussion

We evaluated a new eGFR equation for children under 2 years of age. Using several statistical techniques, we confirmed that the eGFR_under 2_ equation could be useful.

For Japanese children, there are several eGFR equations with surrogate markers, serum Cr [3], cystatin C [7], and β2 microglobulin [8]. The Cr-based eGFR _Uemura_ equation is the most commonly used method for children aged 2–18 years. Cystatin C and β2 microgrobulin based eGFR are not always available for retrospective epidemiologic studies or common clinical practice. Therefore, we developed Cr-based eGFR for children younger than 2 years using eGFR_Uemura_ using a coefficient. The method reported in a previous article [5] is based on the following idea. Physiologically, kidney function gradually increases from birth and reaches adult stage at the age of 2 years. Using the median normal reference values of GFR for each age (months) up to 2 years examined previously, we estimated the percentage of the normal adult GFR that corresponds to and calculated a regression curve using a logarithmic function. As a result, the coefficient was calculated as 0.107 × ln [age (months)] + 0.656. The aim of this study was to evaluate the eGFR _under 2_ equations. We compared the values of eGFR_under 2_, eGFR_Uemura_, and eGFR_Schwartz_ equation using ME, RMSE, *P*_30_, and Bland–Altman analysis. We divided the patients according to age: 13 patients under 2 years of age and 10 patients aged between 1 and 2 years; patient data underwent two analyses. Because eGFR_under 2_ equation is for children under 2 years old, the eGFR_Uemura_ equation is for children aged 2–18 years and eGFR_Schwartz_ is for children aged 1–16 years. Therefore, in the comparison of 10 patients at the age of 1 to 2 years, we only compared eGFR_under 2_ equation and eGFR _Schwartz_. In the analysis of 13 patients, the ME value of eGFR_under 2_ was the smallest among the three equations. The RMSE value and percent of *P*_30_ of eGFR_under 2_ and eGFR_Uemura_ were similar. On the other hand, the ME and RMSE values of eGFR _Schwartz_ were the highest, and the percentage of *P*_30_ was the lowest among the three eGFR equations. In the analysis of 10 patients, the ME and RMSE values of eGFR_under 2_ were lower than that of eGFR_Schwartz_. The percentage of P_30_ of eGFR_under 2_ was higher than that of eGFR_Schwartz_.

This study has several limitations. First, the sampling data was small particular under 1 year of age. Although we collected patients’ data from our three previous studies, there were only 13 among 373 patients (3.5%) under 2 years of age. Further, it is very rare to perform inulin clearance in patients under 2 years of age, thus limiting data collection. In addition to that, there were only two patients of a female under the age of two. However, since there was no gender difference in normal Cr values at younger ages [9], we believed that this result was not affected. Second, to add to the data as much as possible, we also used the patient data when creating the equation of eGFR_Uemura_. Third, we did not exclude preterm infants and low birth weight infant because we had no information for them. We don’t know whether the eGFR_under 2_ equation can be applied to such cases or not.

## Conclusion

We evaluated the eGFR_under 2_ equation for Japanese children aged 2 years or less. The results suggest that eGFR_under 2_ equation could be a useful parameter.
